# Huge Tongue Lipoma: A Case Report

**Published:** 2015-03

**Authors:** Mohammad Ali Damghani, Mohammad Safari

**Affiliations:** 1 *Department of Otorhinolaryngology, Kerman University of Medical Sciences and Health Services, Kerman, Iran.*

**Keywords:** Lipoma, Tongue, Tumor

## Abstract

**Introduction:**

Lipomas are among the most common tumors of the human body. However, they are uncommon in the oral cavity and are observed as slow growing, painless, and asymptomatic yellowish submucosal masses. Surgical excision is the treatment of choice and recurrence is not expected.

**Case Report:**

The case of a 30-year-old woman with a huge lipoma on the tip of her tongue since 3 years, is presented. She had difficulty with speech and mastication because the tongue tumor was filling the oral cavity. Clinical examination revealed a yellowish lesion, measuring 8 cm in maximum diameter, protruding from the lingual surface. The tumor was surgically excised with restoration of normal tongue function and histopathological examination of the tumor confirmed that it was a lipoma.

**Conclusion:**

Tongue lipoma is rarely seen and can be a cause of macroglossia. Surgical excision for lipoma is indicated for symptomatic relief and exclusion of associated malignancy.

## Introduction

Lipomas are the most common benign soft tissue mesenchymal neoplasm; however, they are not common in the oral cavity (1,2) and are usually observed as slow-growing, painless, and asymptomatic masses. It is known that, with continued growth, their size may interfere with speech and mastication (3,4). Oral lipomas can occur in various anatomic sites including the major salivary glands, buccal mucosa, lip, tongue, palate, vestibule, and the floor of the mouth. Various case reports have described lipomas and its variants in several locations (5-7). The buccal mucosa and the tongue are the predominant sites in adults. Some studies show a male preference while other studies show no gender differences (8-10). The tumors are either encapsulated, non-encapsulated, or present in an infiltrating manner. Oral lipoma usually occurs as a solitary lesion. The color is often yellow in tone, depending on the thickness of the overlying mucosa.

***Case Report***

**Fig1:**
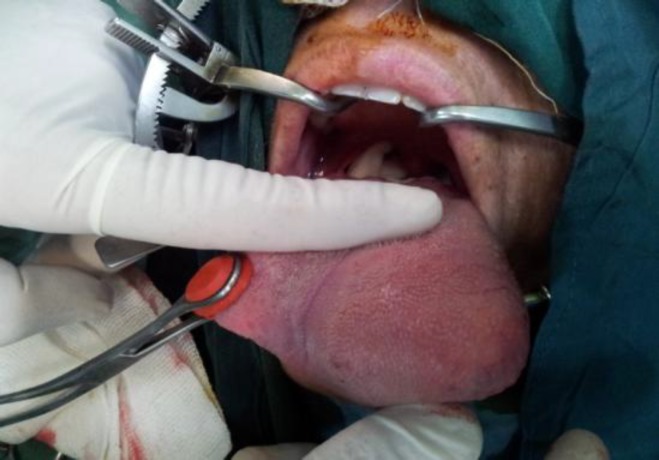
Mass in antrolateral part of the tongue; during palpation, the lesion was observed to be rubbery and compressible.

 A 30-year-old female was presented to the head and neck clinic with a slow-growing mass on the tip of her tongue, which had been present for 3 years. Her speech was not clear due to the bulkiness of the mass and she also had difficulties swallowing. Clinical examination revealed a yellowish lesion measuring 8 cm in diameter protruding from the lingual surface and covered by a mucosa that was rich in vessels. During palpation, the lesion was observed to be rubbery and compressible (Fig.1).

Although the tongue movement was limited, there was no ankyloglossia. Taste and somatic sensation were intact.

**Fig 2:**
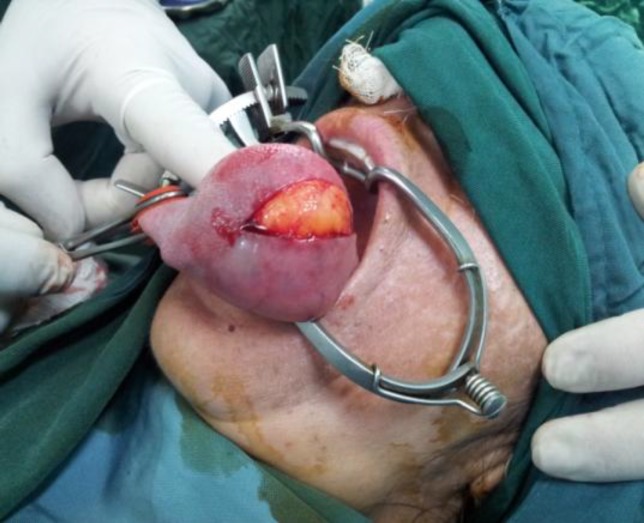
Under general anesthesia, through a longitudinal incision along the edge of the tongue, the tumor was removed

Under general anesthesia, through a longitudinal incision along the edge of the tongue, the tumor was removed (Fig. 2).

**Fig 3:**
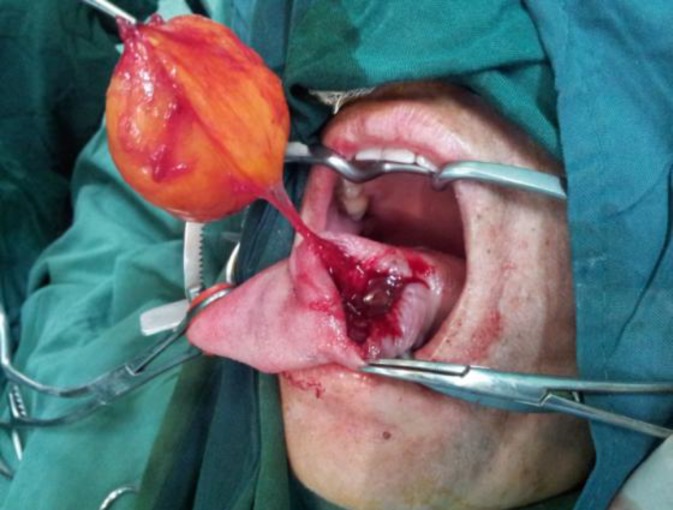
The tumor, which was yellowish in color, 8 cm in diameter, and well-encapsulated, was completely removed

The tumor was yellowish in color and well-encapsulated (Fig.3). The mucosal layers were close together with absorbable sutures obliterating the dead space. The musculature volume of the left side of tongue was decreased.

Histological examination revealed mature adipocytes without cellular atypia. Lipoblast, which is pathognomonic for malignant liposarcoma, was not present.

## Discussion

The majority of tongue tumors are malignant in nature. Lingual lipoma, which accounts for 0.3% of tongue neoplasms, is a benign condition. Similarly, the occurrence in the oral cavity is rare and reported as 2% to 4% of all lipomas (11,12). It is typically described as well- circumscribed, submucosal, with less than 1 cm swelling, and located on the lateral edge of the anterior two-thirds of the tongue surface (13). Microscopically, it is composed of mature adipocytes; however, in 20% of cases, it demonstrates histological variants that include spindle cell lipoma, pleomorphic lipoma, angiolipoma, fibrolipoma, myxoidlipoma, and atypical lipoma. 

In this study the patient was a 30-year-old female with a slow growing mass present since the last 3 years, which measured 8 cm in diameter. The mass was painless but she had difficulties swallowing and tongue movement was impaired; however, taste and somatic sensation was intact. 

In other studies: Chunkitchung reported a 62-year-old man with a 6cm mass in his tongue that was slow growing for 2 years. He had difficulties swallowing large food items. Moreover, his speech was not very clear due to the bulkiness of the mass (14).

Magadum reported a 60-year-old man with a 3cm mass in his tongue, which he had first noticed about 10 years earlier. Because of the absence of pain and bleeding, he was not initially alarmed, but later he complained of masticatory problems (15).

Chidzonga reported a 58-year-old female with an 11cm mass that had been present for 3 years. She had a large “anterior open bite” and slurred speech with the tumor bobbing up and down and in and out of the mouth when speaking. Despite the feeding and breathing difficulties, she was well nourished and not in any particular distress (16). Chandak also reported a 75-year-old man with a mass on the anterior border of the tongue, which he had first noticed 16 years earlier. He had difficulty in mastication and swallowing, and frequently used to wake up from sleep because of obstruction in his airway (17).

Finally Colella reported a 75-year-old man with a 10 cm mass in his tongue from 30 years ago. His speech was not very clear due to the bulkiness of the mass and he had difficulties swallowing (18).

These studies are generally without gender predilection (4,9,10,19-21); however, some studies have shown a male preponderance (13). Lipomas may be observed as solitary or multiple lesions, such as Gardner’s or Bournville’s syndrome (19,22), or as macroglossia (22-26) or lipomatosis (27).

Their clinical course is usually asymptomatic until they grow to large sizes (19,22). In the present case, the large size interfered with speech and mastication, similarly to a case reported by Gray and Baker (22). Large tumors have been shown to cause dentofacial deformities and anterior open bite (9,10). On rare occasions, the infiltration is so extensive that it can cause muscle dysfunction or sensory changes due to pressure on nerve trunks. Pain is rarely severe (28,29). The average duration of the lipoma before excision is 3.2 years with a range of 6 weeks to 15 years (21). The usual range in size is 0.5 to 8 centimeters (21). The present case was 8 centimeters in diameter.

The differential diagnosis includes well-differentiated liposarcoma, ranula, dermoid cyst, thyroglossal duct cyst, ectopic thyroid tissue, pleomorphic adenoma, and mucoepidemoid carcinoma angiolipoma, fibrolipoma, and malignant lymphoma (19,23-26). The definitive diagnosis is by microscopic examination, which shows adult fat tissue cells embedded in a stroma of connective tissue and surrounded by a fibrous capsule (26). Lipoma has a characteristic radiographic appearance. On CT scan it shows a high density from 83 to 143 Hamsfield units with well or poorly defined margins depending on the capsule (19). Ultrasonography shows a lesion, which is round or elliptical in shape with an intact or mostly intact capsule (30). 

Surgical excision is the most common form of treatment (19,21). Recurrence is reduced by wide surgical excision while preserving the surrounding structures. Well-encapsulated lipomas, as the present case, easily shell out with no possibility of recurrence or damage to the surrounding structures. It is still advisable to excise them with a little cuff of surrounding normal tissue to prevent recurrence while still conserving surrounding structures (11). 

## Conclusion

Tongue lipoma is rarely seen and can be a cause of macroglossia. Surgical excision for lipoma is indicated for symptomatic relief and exclusion of associated malignancy
